# Supplementary material for: Reference Values for Fitness Level and Gross Motor Skills of 4–6-Year-Old Chilean Children
Source: Int J Environ Res Public Health. 2020 Jan 28;17(3):797. doi: 10.3390/ijerph17030797 (PMC7038087; doi:10.3390/ijerph17030797)
Supplement: Supplementary file 1 [file ijerph-17-00797-s001.pdf]

International Journal of
Environmental Research and Public Health

MDPI

Supplementary Materials: Reference Values for Fitness Level and Gross Motor Skills of 4–6-Year-Old Chilean Children

Fitness level

20-m shuttle run test: The participants had to run back and forth between two separate lines 20 meters with an audio signal. The test finished when the child could not reach the concurrent end lines with the audio signal on two consecutive occasions or when the child stopped due to exhaustion.

Standing long jump test: Lower body muscular strength was assessed by the standing long jump test. This test consists in jumping as far as possible with feet together (separate from each other approximately at the shoulder's width) and remaining upright.

Handgrip dynamometry: Upper body muscular strength was assessed by the handgrip strength test using a dynamometer. The children squeeze gradually and continuously for at least two or three seconds, performing the test twice (alternately with both hands). The elbow should extended and avoid contact of any other part of the body with the dynamometer, except the hand that is measured. The optimal grip span was set at 4.0 cm.

4x10m shuttle run: This test consisted of running and turning as fast as possible between two parallel lines (separated by 10 meters) drawn on the floor, covering a distance of 40m. To simplify this test, two evaluators were placed at both ends and the participants had to touch the evaluator hand (placed behind the line) and return to maximum speed. The best of two attempts (seconds) was recorded.

Sit and Reach test: The subjects take off their shoes and sit on the test equipment with their knees fully extended and their feet shoulder-width apart. The feet should be flat against the final board. The arms extend forward with the hands placed on top of each other to perform the test. The subject comes straight forward, palm down, along the measurement scale and maintains the maximum range position. The test equipment consists of a specially constructed box with a measuring scale.
